# Radical Scavenging and Antioxidant Activity of *Anthyllis Vulneraria* Leaves and Flowers

**DOI:** 10.3390/molecules23071657

**Published:** 2018-07-07

**Authors:** Manel Ouerfelli, Leila Bettaieb Ben Kâab, María Pilar Almajano

**Affiliations:** 1 Research Unit “Nutrition et Métabolisme Azotés et Protéines de Stress” (UR/ES-13-29), Biology Department, Faculty of Sciences of Tunis (FST), University of Tunis El-Manar (UTM), University Campus of Tunis El-Manar, 2092 Tunis, Tunisia; bio.m.0105.ouerf@gmail.com (M.O.); leila.bk@planet.tn (L.B.B.K.); 2 Chemical Engineering Department (DEQ), School of Industrial Engineering of Barcelona (ETSEIB), Universitat Politècnica de Catalunya (UPC), Av, Diagonal 647, 08028 Barcelona, Spain

**Keywords:** *A. vulneraria*, phenolic compounds, antioxidant activity, lipid oxidation, emulsion, beef patties

## Abstract

The main targets of this work were to determine the phenolic content of *Anthyllis vulneraria* (*A. vulneraria*) leaves and flowers and to evaluate their antioxidant activity. Total polyphenols and flavonoid content (TPC and TFC, respectively) were determined. Antioxidant capacity was evaluated by the Ferric Reducing Antioxidant Power (FRAP), the Oxygen Radical Absorbance Capacity (ORAC), the Trolox Equivalent Antioxidant Capacity (TEAC) and the diphenyl picrylhydrazyl (DPPH) assays, and by the analysis of primary and secondary oxidation products in oil-in-water emulsions and in raw beef patties during storage. The results revealed that the flowers of the *A. vulneraria* contained the highest content of total polyphenols and flavonoids and extracts from these tissues exhibited the strongest antioxidant activity, as they were more effective at retarding lipid oxidation in oil-in-water emulsions and raw beef patties than extracts from the leaves which had a potent antioxidant effect only at the beginning of the oxidation process. The results of this study allowed us to obtain a deep knowledge about the properties of *A. vulneraria* and confirmed the possibility of using its biologically active extracts in the food, cosmetic and pharmaceutical industries.

## 1. Introduction

In recent years, the search for novel natural and functional extracts from medicinal plants has attracted a growing amount of interest because of the rich content of bioactive molecules such as phenolic compounds, vitamins and proteins [1] present in the different parts of the plants [2]. These bioactive compounds are gaining a very important role in various industrial fields such as the pharmaceutical, cosmetics and food industries thanks to their antioxidant, antimicrobial and anticancer properties [3] allowing them to retard the development of several fatal diseases caused by Reactive Oxygen Species (ROS) [4].

Phenolic compounds are secondary metabolites occurring with an unequal qualitative and quantitative distribution in plants. They include an important variety of compounds such as phenolic acids, flavanols, anthocyanins, stilbenes, etc., that vary in their basic structure but possess an aromatic ring bearing one or more hydroxyl groups [5]. These compounds play a crucial antioxidant role through different mechanisms of action, by scavenging free radicals, quenching ROS, inhibiting oxidative enzymes and chelating transition metals [6,7]. This is one of the reasons why many studies have focused on obtaining natural antioxidants from plants as alternatives to synthetic antioxidants, such as butylated hydroxytoluene (BHT) and butylated hydroxy-anisole (BHA), which may present harmful effects on human health in the long term [8]. Emulsions and raw meat are good models to study the antioxidant effects of herbs [9]. 

The benefits of phenolic compounds are not limited to their antioxidant property, but they have also been reported to protect the human organism from several chronic and degenerative health disorders and diseases due to their anticarcinogenic, antimutagenic, and antimicrobial properties that have been attributed to their antioxidant activity [10,11]. Despite advanced research and the discovery of treatments, many infectious and cancerous diseases remain a global problem causing deaths worldwide. Therefore, numerous studies have been conducted to identify new antimicrobial and anticancer agents from herbs [12,13]. 

Among the wild plant families most exploited for their secondary metabolites, the Fabaceae family, with more than 18,000 identified species, is considered as an excellent source of proteins, dietary fibers and various phytochemicals with important health benefits, such as anticancer, antimicrobial, and anti-obesity effects [14,15]. 

*Anthyllis vulneraria* L. (*Anthyllidis flos*, *herba*; *Papilionaceae*) [16], is a Mediterranean medicinal plant that belongs to the Fabaceae family and is common in the pastures of mountainous regions in Europe, North Africa, South Africa and Southeast Asia [17]. Ethanolic extracts of *A. vulneraria* have been used in traditional medicine to inhibit the multiplication of human herpesvirus 1 and poliovirus 2 in cell culture [18]. Its flowers were used to treat wounds, high blood pressure, heart failure, portal hypertension, vomiting, inflammation, acne and disturbances of metabolism, and to purify the body by promoting the elimination of toxins. They were also used to heal mouth and throat pain and to enhance hair growth [16,19]. Moreover, its leaves contain several bioactive substances such as phenolic acids, flavonoids, carotenoids, tannins and saponins [20].

The main targets of this study were to determine the total polyphenol and flavonoid contents of the leaf and flower extracts from *A. vulneraria* collected from the northeast of Tunisia, to evaluate their antioxidant activity by the ORAC, FRAP, TEAC and DPPH assays and to assess their usefulness in oil-in-water emulsions and in raw beef patties. 

## 2. Results and Discussion

### 2.1. Total Polyphenol and Flavonoidcontents

The TPC and the TFC of the *A. vulneraria* leaf and flower extracts in 50% aqueous ethanol were determined and the results obtained with significant differences between the samples (*p* < 0.05) are shown in Table 1. 

The flower extract of *A. vulneraria* contained 62% higher total polyphenol content and 67% higher flavonoid content than the leaf extract.

Several authors have determined the contents of total polyphenols and flavonoids in *A. vulneraria* from different tissues. Godevac et al. [19] studied the chemical composition of *A. vulneraria* and reported similar total polyphenols content of 108.1 and 79.34 mg GAE/g dry plant in the flower extracts of *A. vulneraria* from Montenegro and Serbia, respectively. However, Tusevski et al. [21] found that the extracts of the aerial part of *A. vulneraria* from Macedonia contained lower contents of total polyphenols and flavonoids than those obtained in this study with values 12.02 mg GAE/g DW and 2.22 mg CE/g DW, respectively. 

Many other medicinal plants belonging to the Fabaceae family, including *Albizia julibrissin, Desmodium caudatum, Lespedeza bicolor, L. cuneata, L. maximowiczii, Pueraria lobata, Robinia pseudoacacia, Sophora flavescens, S. japonica* and *Erythrina stricta Roxb* contain high contents of phenolic compounds [22]. Skowyra and Gallego [23] reported high contents of total polyphenols and flavonoids in *Caesalpinia spinosa* pods estimated at 460.2 mg GAE/g dry plant and 2.93 mg CE/g dry plant, respectively. Gallego et al. [2] determined the phenolic compounds in the leaf extracts from Tara (*Caesalpinia spinosa*) and Mysore thorn (*Caesalpinia decapetala*) and found that the *C. spinosa* leaf extract extracted with 50% aqueous ethanol contained higher contents of total polyphenols compared with our results, while *C. decapetala* containeda lower polyphenol content of 63.8 mg GAE/g dry plant. 

Differences in the distribution of the polyphenols and flavonoids arise from various factors that can be biological e.g., the part analyzed and the vegetative stage of the plant [24] and technical such as the extraction method, the solvents and their concentrations [25,26] and there are also differences in the structure and properties of the phenolic compounds present in the different samples analyzed [27].

Recent studies confirmed the key role that the polarity of the extraction solvent plays in extracting phenolic compounds from plant materials. Cheung et al. [28] and Ye et al. [29] found that aqueous alcohols are more effective solvents for extraction of phenolics from the florets of Sunflower and Moringa. Moreover, ethanol is preferred to other solvents and wildly used as a solvent to extract phenolics from plants because it is considered as a GRAS solvent (Generally Recognized As Safe) that can be used safely for food and industrial products without fear of toxicity [30,31].

### 2.2. Antioxidant Activity

#### 2.2.1. Free Radical Scavenging Activity

The results of the antioxidant activity of the *A. vulneraria* leaf and flower extracts are presented in Table 2.

Significant differences in the antioxidant activity determined by the different methods were found (*p* < 0.05). The flower extract showed the best antioxidant activity with 74%, 67%, 73% and 94% higher values than the leaf extract determined by the FRAP, ORAC, TEAC and DPPH assays, respectively. In previous studies, the antioxidant activity of *Anthyllis* extracts has been determined using the TEAC method. Godevac et al. [19] obtained lower TEAC values of 0.448 mM Trolox/g dry plant in the flower extract of *A. vulneraria* from Serbia and 0.909 mM Trolox/g dry plant in the flowers extract of *A. aurea*. High antioxidant activity has also been found in different extracts from members of the *Fabaceae* family, for instance the extracts from the pods of *Caesalpinia cacalaco* [32], *Acacia pennatula* [33], extracts from the flowers and roots of *Onobrychis armena* [34] and extracts from the seeds of *Trigonella foenum-graecum* [35].

#### 2.2.2. Effects of the *A. vulneraria* Extracts on the Oxidative Stability of Emulsions

**Figure 1:**
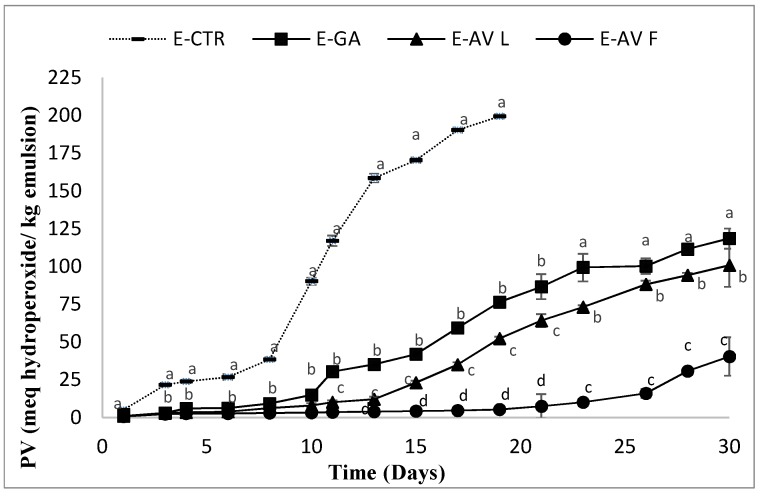
Peroxide values (PV) of emulsions containing *A. vulneraria* leaf and flower extracts during storage. E-CTR (Control emulsion sample), E-GA (Galic acid emulsion sample), E-AVL (*A. vulneraria* leaf emulsion sample), and E-AVF (*A. vulneraria* flower emulsion sample). Error bars represent the standard deviation (*n* = 3) and different letters in the same day indicate significant difference between samples at *p* < 0.05.

Several studies relevant to the food industry have investigated the antioxidant effects of extracts containing phenolic compounds on lipid oxidation in food systems. In the present study, the effect of the *A. vulneraria* leaf and flower extracts, extracted with 50% aqueous ethanol, on lipid oxidation in oil-in-water emulsion was investigated during 30 days of storage at 33 ± 1 °C. The evolution of lipid oxidation in the emulsion samples was determined by monitoring the changes in the peroxide values (PV), the pH and the Thiobarbituric acid reactive substances (TBARS) values of the emulsions during the storage time. The evolution of primary oxidation (PV) over the storage time is illustrated in Figure 1. 

To ensure a good quality of consumable products containing fats, the maximum peroxide value was estimated by the Codex Alimentarius to be 10 meqhydroperoxide/kg of oil [26]. Considering this value as a measure of emulsion stability, the formation of hydroperoxides was significantly swiftest in the E-CTR sample, which showed an increase of the oxidation values from the first day of storage. The E-GA and the E-AVL remained stable against lipid oxidation for 8 to 11 days respectively, and then exhibited a faster oxidation, whereas the E-AVF sample was stable against lipid oxidation until 23 days. After 19 days of storage, the PV of the E-CTR sample reached a maximum of hydroperoxide content with 199.37 meq hydroperoxide/kg emulsions. At the end of the storage period (30 days), the E-GA and E-AVL samples had also deteriorated with maximum peroxide values of 188.47 and 100.78 meq hydroperoxide/kg emulsion, respectively. The order of the oxidation stability of the different emulsions was consistent with the order of the phenolic contents and the antioxidant assay values. The E-AVF sample presented the best protective effect against the formation of the primary oxidation products, followed by the E-AVL then the E-GA.

The *A. vulneraria* extracts were more effective at retarding lipid oxidation compared with several previous research reports of the antioxidant effect of herbs on protecting emulsions from lipid oxidation. Gallego et al. [2] determined the peroxide values of emulsions containing 10% of purified sunflower oil containing extracts from *Caesalpinia spinose* (*C. spinose*) and *Caesalpinia decapetala* (*C. decapetala*) at a concentration of 0.5% during 33 days of storage at 33 °C. These authors reported that their extracts reached the limit allowed for products containing edible fats (10 meg/kg oil) after 18 days of storage and were successful in slowing down lipid oxidation during the whole storage period with peroxide values of 6.7 and 18.2 meg hydroperoxides/kg emulsion, respectively. In another study conducted by Skowyra et al. [36], the *Artemisia annua* extract was effective also in slowing down the formation of hydroperoxides and reached 10 meg/kg oil after 28 days. An amount of 0.5% w/w of *Gentiana lutea* extract was able to retard lipid oxidation throughout storage with samples reaching the limit for fat products after 10 days [37]. An extract from Pineapple Waste was effective at retarding lipid oxidation according to the study of Segovia and Almajano [38].

**Figure 2:**
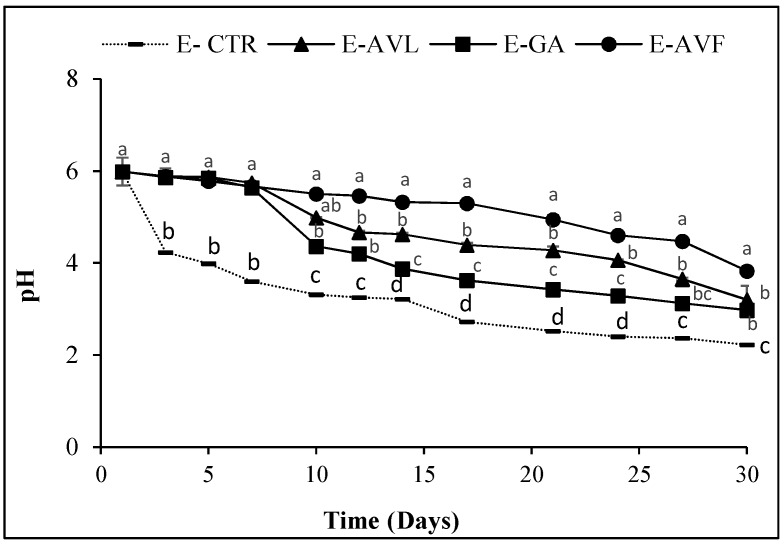
pH evolution during 30 days of study. E-CTR (Control emulsion sample), E-AVL (*A. vulneraria* leaf emulsion sample), E-GA (Galic acid emulsion sample), E-AVF (*A. vulneraria* flower emulsion sample). Error bars represent the standard deviation (*n* = 3) and different letters in the same day indicate significant difference between samples at *p* < 0.05.

The oxidation of lipids causes qualitative and nutritional alterations such as rancidity, loss of vitamins and even toxicity due to lipid peroxidation products like peroxides and aldehydes. The hydroperoxides are the main primary products generated by lipid oxidation. However, their instability causes their easy decomposition into secondary compounds, resulting in the appearance of aldehydes and acidic oxidation products [39]. For this reason, the pH of the different samples was also determined in our study as an indicator of emulsion lipid oxidation. The evolution of the peroxide values (Figure 1) and the pH values (Figure 2) were inversely proportional.

All the samples started with an initial average pH value of 5.98. The first decrease of the pH values was observed in the E-CTR from 5.98 to 3.89 after only three days of storage. The E-AVF was the only sample that remained stable at around 5 for more than 17 days before decreasing to 3.82 at the end of the storage time. The E-GA had a similar behavior to the E-AVL. Their pH remained stable at around 5.51 for 8 days, then decreased to a pH value of 2.97 and 3.20, respectively, at the end of the storage time. The change and fall of the pH during lipid oxidation is due to the decrease in the efficacy of the phenolic compounds present in the extracts to prevent the formation of oxidation products including hydroperoxides and their acidic degradation products [40]. Decker et al. [41] showed that pH also affects the rate of oxidation by different mechanisms. For example, pH can affect the redox state of metals and the activity, solubility, stability, and chelation capacity of antioxidants, and it can influence the distribution of antioxidants between the aqueous and lipid phases. 

**Figure 3:**
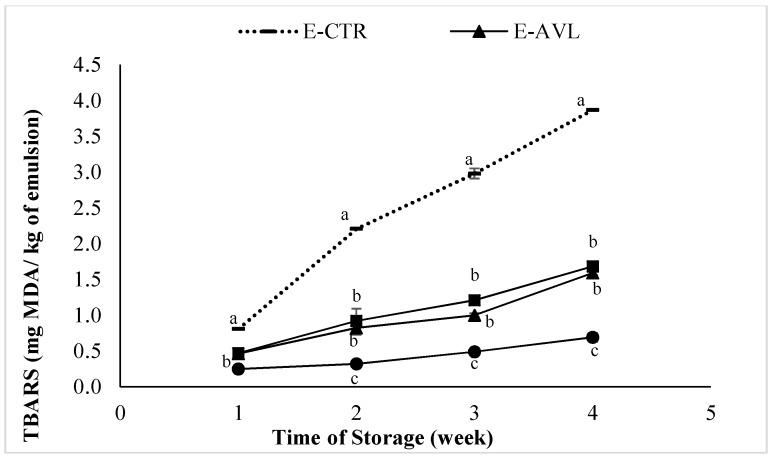
Changes in Thiobarbituric Acid Reactive Substance (TBARS) values of the emulsion containing *A. vulneraria* leaf and flower extracts during storage. E-CTR (Control emulsion sample), E-AVL (*A. vulneraria* leaf emulsion sample), E-GA (Galic acid emulsion sample), E-AVF (*A. vulneraria* flower emulsion sample). Error bars represent standard deviation (*n* = 3) and different letters in the same day indicate significant difference between samples at *p* < 0.05.

The degradation of hydroperoxides into second compounds leads to the formation of aldehydes. Malondialdehyde (MDA) is one of the main secondary products responsible for the bad flavor, the rancid odor and undesirable taste of oxidized fats [42]. In the present study, this compound was monitored by the measurement of the TBARS values (Figure 3).

#### 2.2.3. Effects of Powdered *A. vulneraria* on Raw Meat Oxidative Stability

##### Lipid Oxidation and Changes in pH Values

**Figure 4:**
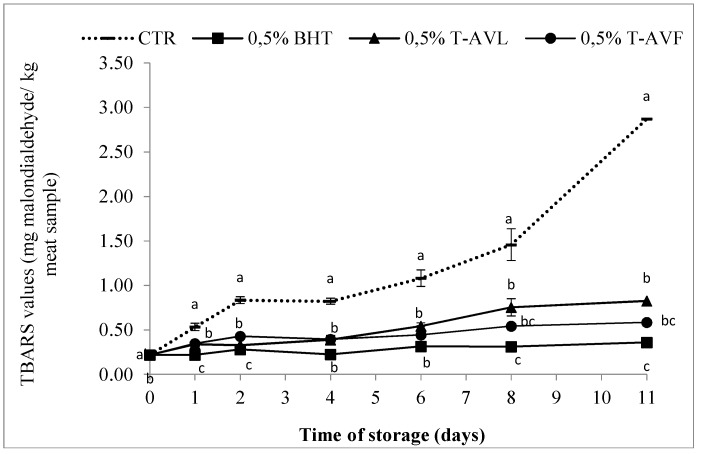
Thiobarbituric Acid Reactive Substance (TBARS) values of raw beef patties formulated with powdered leaves and flowers of *A. vulneraria* during storage at 4 ± 1 °C. CTR (Control sample without antioxidant), 0.5% BHT (treatment with 0.5% synthetic antioxidant (Butylated hydroxytoluene)), 0.5% T-AVL (treatment with 0.5% *A. vulneraria* leaf), 0.5% T-AVF (treatment with 0.5% *A. vulneraria* flower). Results represent the mean of three replicates (*n* = 3) and are expressed as mean value ± SD; different letters in the same day indicate significant difference between samples at *p* < 0.05.

The effect of powdered *A. vulneraria* leaves and flowers on the lipid oxidation of raw beef patties during 11 days of storage (DOS) at 4 ± 1 °C was evaluated and the results are shown in Figure 4. 

The TBARS values increased continually (*p* < 0.05) despite the low temperature of storage from 0.22 ± 0.01 mg MDA/kg ground beef sample (initial value) to 2.87 ± 0.18, 0.36 ± 0.02, 0.83 ± 0.10 and 0.59 ± 0.03 in the control sample, 0.5% BHT, 0.5% T-AVL and 0.5% T-AVF, respectively. According to Leygonie et al. [43], storage time and temperature have a significant influence on lipid oxidation in raw beef patties. Cool temperature storage is not completely effective in preventing meat from deterioration and the freezing and thawing of raw meat is an incentive to accelerate lipid oxidation, and thus the formation of secondary products. 

The results obtained showed that natural antioxidants have a significant influence (*p* < 0.05) on the development of lipid oxidation. The TBARS values of all the treated samples including 0.5% BHT, 0.5% T-AVL and 0.5% T-AVF, were significantly (*p* < 0.05) lower than for the CTR sample. Leaf powder contributed stronger lipid stability than flower powder during storage as shown by the lower TBARS values. This can be explained by the higher content of natural polyphenol, which are responsible for strong antioxidant activity, due to their ability to neutralize and eliminate free radicals [44]. The effectiveness of natural antioxidants for limiting lipid oxidation and extending the shelf-life of meat products was demonstrated in a previous study [45], which showed that Tara pod powder, when battered with pork meat, had high antioxidant activity and retarded lipid oxidation during chilled storage. The results obtained are consistent with those reported by Azman et al. [46] who found that 0.1% *Convolvulus arvensis* samples were more stable (*p* < 0.05) than samples treated with 0.1% BHT. It was also reported that a by-product, avocado waste, was also efficient in inhibiting rancidity deterioration of beef and pig meat [43,44].

Changes in pH values during storage of raw beef patties formulated with powdered leaves and flowers of *A. vulneraria* are shown in Table 3.

The pH values were significantly different between the different samples (*p* < 0.05) but not in the same sample during 11 days and increased continually with storage time from 5.86 ± 0.03; 5.69 ± 0.01; 5.76 ± 0.01 and 5.71 ± 0.01 on the first day to 6.19 ± 0.03; 5.78 ± 0.02; 6.01 ± 0.05 and 5.86 ± 0.02 on day 11 (last day) for the CTR, 0.5% BHT, 0.5% T-AVL and 0.5% T-AVF, respectively. After 11 days of refrigerated storage, the control had the highest pH values (6.19 ± 0.03), whereas the 0.5% BHT sample had the lowest pH value (5.78 ± 0.02). T-AVF had lower pH values compared with the control and T-AVL. Muela et al. [47] argued that degradation of proteins in muscle tissues, due to microorganisms, results in the production and accumulation of ammonia, amines and other basic substances that are responsible for the increase in the pH of the meat and its products. 

##### Color Changes

The color of meat is a good sign of its quality and freshness, which are considered amongst the most influential factors that affect consumer acceptance and purchasing decisions [48]. The effect of the addition of powdered *A. vulneraria* leaves and flowers to raw beef patties on their surface color is shown in Table 4.

The most important color parameter to evaluate the consumer preference of meat is the Redness (a*) value since its decrease alienates consumers [49]. In this study, Redness (a*) values decreased in all the samples of patties during the period of storage at 4 ± 1°C. Compared with 0.5% T-AVL, Redness (a*) values of 0.5% T-AVF sample were significantly higher (*p* < 0.05) but slightly lower than the 0.5% BHT sample. Redness (a*) values of the 0.5% T-AVF sample decreased from 49.46 ± 0.37 in the first day of analysis to 25.74 ± 1.03 in day 11. The lowest values of Redness were recorded in the control sample, which were considerably (*p* < 0.05) decreased during chilled storage from 36.67 ± 2.37 in the first day to 24.89 ± 1.23 in the last day. A similar trend for fresh beef patties containing olive cake powder during storage at 4 °C (±1) was reported [50]. According to Mancini and Hunt [51], the reduction of Redness (a*) values during storage is probably due to the oxidation of myoglobin and the formation of metmyoglobin.

Besides Redness (a*) values, powdered *A. vulneraria* leaves and flowers enhanced the Yellowness (b*) values of raw beef patties. The Yellowness (b*) values of non-treated (CTR) and treated (0.5% BHT, 0.5% T-AVL and 0.5% T-AVF) samples decreased significantly (*p* < 0.05) during refrigerated storage. The lowest Yellowness (b*) values were observed in the stored control samples and values increased with the addition of *A. vulneraria* leaf and flower powder. Higher values were recorded in 0.5% T-AVF samples than in 0.5% T-AVL samples and were close to the values recorded in 0.5% BHT samples. An increase in Yellowness (b*) values was observed also in raw ground pork patties containing *Moringa oleifera* leaves [52]. Eventually, as shown in Table 4, the Lightness (L*) values of each sample decreased significantly over time of storage.

The mean Lightness (L*) values of the control sample were lower than those of the treated samples. The addition of powdered *A. vulneraria* leaves and flowers had a significant effect when compared with the control. In previous studies conducted by Esmer et al. [53] and Gallego et al. [49], slight changes in Lightless (L*) values in meat during chilled storage were reported:

##### Antioxidant Capacity Determined by the FRAP Assay

**Figure 5:**
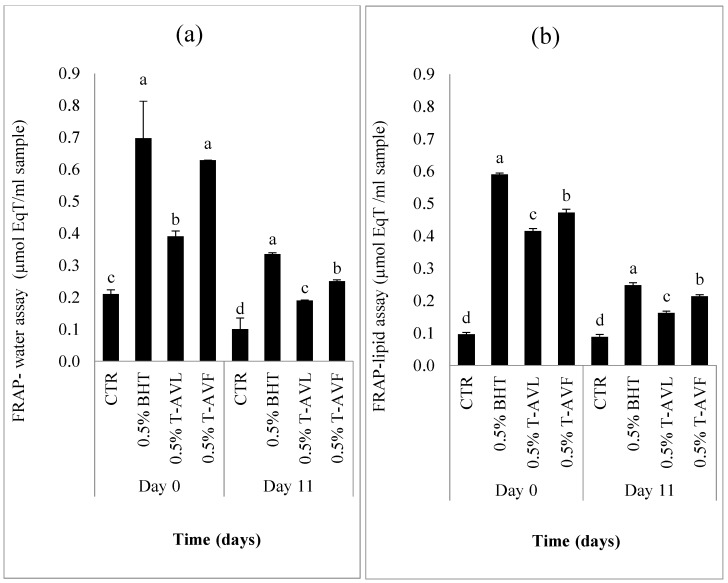
Antioxidant Capacity. (AOC) measurements by FRAP water (**a**) and FRAP lipid (**b**) assays of each beef patty sample formulated with powdered leaf and flower of *A. vulneraria.* CTR (Control sample without antioxidant), 0.5% BHT (treatment with 0.5% synthetic antioxidant (Butylated hydroxytoluene)), 0.5% T-AVL (treatment with 0.5% *A. vulneraria* leaf), 0.5% T-AVF (treatment with 0.5% *A. vulneraria* flower). Results represent the mean of three replicates and are expressed as mean value ± SD; different letters in the same day indicate significant difference between samples at *p* < 0.05.

The results of the antioxidant capacity assay determined by the FRAP-water and FRAP-lipid assays on day 0 (initial day) and day 11 (last day) are summarized in Figure 5a and Figure 5b, respectively.

Each sample was tested by the hydrophilic and lipophilic antioxidant activity assays. The significantly highest values (*p* < 0.05) of the hydrophilic antioxidant assay were recorded in the 0.5% BHT sample (0.34 ± 0.01), followed by the 0.5% T-AVF (0.25 ± 0.01) and then 0.5% T-AVL samples (0.19 ± 0.02), while the CTR had the lowest antioxidant activity (0.10 ± 0.01) µmol eq Trolox/mL sample (Figure 5a). For the hydrophilic antioxidant assay, the significantly highest values (*p* < 0.05) of the lipophilic antioxidant assay were recorded in the 0.5% BHT sample (0.25 ± 0.02), followed by the 0.5% T-AVF (0.21 ± 0.02) then 0.5% T-AVL samples (0.16 ± 0.05) while the control represented the lowest antioxidant activity (0.09 ± 0.01) µmol eq Trolox/mL sample (Figure 5b). All the samples, including the control, had higher hydrophilic FRAP values than lipophilic ones with no significant difference between the same samples in each FRAP assay. The results obtained in this study were higher than those of the *Caesalpinia decapetala* extract showing that the *C. decapetala* can be a good source of natural antioxidant since it had higher antioxidant capacity determined with hydrophilic and lipophilic FRAP assays and that hydrophilic FRAP values are higher than lipophilic values [49]. Generally, antioxidants can be classified into two different groups, namely lipophilic antioxidants (tocopherols and carotenoids) and hydrophilic antioxidants (ascorbic acid and the majority of phenolic compounds), which contribute to a high antioxidant capacity, enabling the meat products treated with natural antioxidants to be protected against oxidation.

##### Sensory Characteristics

In the present study, a triangle test was used to know if the cooked beef patties formulated with powdered leaves (T-AVL) and flowers (T-AVF) of *A. vulneraria* were identical to the control beef patties (without natural or synthetic antioxidants). Results presented in Table 5 show the sensory evaluation of the beef patties samples.

In fact, 23 assessors from 25 agreed that T-AVL samples had a different taste compared to the control samples. Furthermore, 23 assessors from 30 agreed also that T-AVF samples had a different taste than the control samples. According to the table used to interpret the triangular test results, if the number of assessors is 23 and 30 and the number of correct answers is 15 and 19 respectively, the level of significance is equal to 0.1%. Thus, we conclude from this result that there is a significant difference between T-AVL/T-AVF samples and the control samples. In other words, the reformulated beef patties are significantly different from the original ones.

## 3. Materials and Methods 

### 3.1. Reagents and Chemicals

Alumina; aluminum chloride (AlCl_3_), ethanol (EtOH), iron(II) chloride (FeCl_2_), ferric chloride hexahydrate (Cl_3_Fe·6 H_2_O), gallic acid (GA), methanol (MeOH), potassium persulfate (K_2_S_2_O_8_), sodium carbonate (Na_2_CO_3_), Trolox, tween, 2-thiobarbituric acids (TBA), dimethyl sulfoxide (DMSO), sodium dodecyl sulfate (SDS), 2,4,6-tripyridyl-s-triazine (TPTZ), 2,2′-azo-bis-2-amidinopropane hydrochloride (AAPH); 2,2′-azino-bis-3-ethylbenzothiazoline-6-sulphonic acid (ABTS), 2,2-diphenyl-1-picrylhydrazyl (DPPH) were purchased from Sigma-Aldrich Química S.A. (Madrid, Spain). 

Acetic acid, acetone, ammonium thiocyanate (NH_4_SCN), fluorescein, Folin–Ciocalteu reagent, hydrochloric acid (HCl), phosphate buffered saline (PBS), quercetin, trichloroacetic acid (TCA) were acquired from Panreac Química S.L.U (Barcelona, Spain). 

### 3.2. Spectrophotometric Measurements

Spectrophotometric measurements were performed using FLUOstar^®^ Omega (Ortenberg, Germany), a multimode micro-plate reader with five detection modes using an ultra-fast UV/Vis spectrometer. The spectrophotometer was purchased from BIOGEN Científica, S.L. (Madrid, Spain).

### 3.3. Determination of Phenolic Compounds and Free Radical Scavenging Capacity

#### 3.3.1. *A. vulneraria* Samples and Extraction Procedure

The *A. vulneraria* was harvested at the beginning of April, corresponding to the flowering period, from the mountains of Zaghouan situated in the northeast of Tunisia characterized by a semi-arid climate. The leaves and flowers were separated and dried in air during two weeks, until the achievement of constant weight, and then were ground using a blender. The homogenous powder obtained was stored in amber glass bottles at room temperature for later use. The different extracts were prepared by mixing and stirring 0.25 g of each vegetable matter (leaves and flowers) in 5 mL of 50% ethanol: water (*v*/*v*). The mixtures obtained were centrifuged at 3723 g (Orto Alresa Mod. Consul, Ortoalresa, Ajalvir, Madrid, Spain) for 10 min and then each supernatant was filtered using Whatman filters N°4 and stored at 4 °C.

For all parameters studied below, samples were analyzed in triplicate. 

#### 3.3.2. Total Polyphenol Content (TPC)

TPC was determined using the method based on the Folin–Ciocalteu reagent [38]. Briefly, 20 µL of each diluted sample (1:10, *v*:*v*) were mixed with 80 µL of Folin–Ciocalteu reagent (2N), 80 µL of Na_2_CO_3_ 20%. After mixing for 2 min and incubation at room temperature for 1 h in darkness, the absorbance was measured at 765 nm against a blank, where extracts were replaced by Milli-Q water. The absorbance was measured at 765 nm and Gallic acid (100–1700 µM, R^2^ = 0.992) was used as the standard for the calibration. The results were expressed as mg GAE/g dry plant.

#### 3.3.3. Total Flavonoid Content (TFC)

TFC was determined following the method of Pękal and Pyrzynska [54] with some modifications. An aliquot of 150 µL of each sample was allowed to react with 50 µL of AlCl_3_ (20 mg/mL in acetic acid 5% prepared with MeOH 3:1 ratio). After 30 min of incubation in darkness, the absorbance was measured at 405 nm and the measurements were compared to a calibration curve prepared with Quercetin (50–500 µM, R^2^ = 0.998). Results are expressed as mg QE/g dry plant.

#### 3.3.4. Ferric Reducing Antioxidant Power (FRAP) Assay

FRAP method was carried out as described by Skowyra et al. [36]. In short, a suitable dilution of the *A. vulneraria* extracts was allowed to react with the FRAP reagent and incubated at 37 °C. FRAP reagent was prepared with acetate buffer (300 mM; pH 3.6), 2.4.6. Tri-Pyridyl-5-Triazine (10 mM in HCl, 40 mM) and FeCl_3_ (20 Mm), which were all mixed in the ratio 10:1:1 (*v*/*v*/*v*), respectively. The absorbance was recorded at 593 nm and the FRAP value was determined from a calibration with Trolox (3–20 µM, R2 = 0.989) and expressed as mM Trolox/g dry plant.

#### 3.3.5. Oxygen Radical Absorbance Capacity (ORAC) Assay

ORAC assay was carried out at 37 °C [38]. Shortly, 40 µL of each diluted sample (1:100; *v*:*v*) was mixed with 120 µL of Fluorescein (0.01 mM) and agitated for 2 min.. An initial reading of 2 min at an excitation wavelength of 485 nm was taken, and then 40 µL AAPH (0.3 M) was added to the mixture and the measurement was continued for 3 h at a wavelength of 535 nm. The decrease in fluorescence over time was quantified as area according to Equation (1):

(1) $$\;AUC\;=\;\frac{\left(0.5\;+\;\sum_{t_{c}}^{N_{c}}f_{n}\right)}{f_{i}}$$

where AUC represents the area under the sample curve in the well, *f_i_* represent the fluorescence reading at the initiation of the reaction, f*_n_* represent la the last measurement, N_c_ represent the number of cycles and t*_c_* represent the time of each cycle (2 min).

To calculate the ORAC value, a calibration curve was prepared using Trolox at different concentrations ranging from 0.5 to 14.78 mg Trolox/L. Equation (2) below is specific for determining the decrease in fluorescence at the sample level:

Decrease in fluorescence = AUC − AUC_Bl_ (2)

where AUC_Bl_ expresses the area under the blank curve. ORAC values were expressed in mM Trolox/g dry plant mass.

#### 3.3.6. Trolox Equivalent Antioxidant Capacity (TEAC) Assay

TEAC assay was performed according to the procedure followed by Gallego et al. [26]. ABTS radical solution was prepared with ABTS radical cation (7mM) and K_2_S_2_O_3_ (24.24 mM) and diluted with 10 mM PBS (pH 7.4) to be adjusted to an absorbance of 0.72 then incubated at 30 °C. To perform the assay, 200 µL of the ABTS radical solution was mixed with 20 µL of each sample. The absorbance was measured at 734 nm for 20 min. The final results were taken after 5 min of the absorbance reading. The TEAC values were determined from a Trolox calibration curve with final concentrations ranging from 1 to 10 µM and R^2^ = 0.998. The results are expressed as mM Trolox/g dry plant.

#### 3.3.7. Diphenyl Picrylhydrazyl (DPPH) Assay

The ability of the *A. vulneraria* extracts to scavenge DPPH radicals was assessed by the method of Shalaby and Shanab [55]. An aliquot of each extract was added to 5.07 mM DPPH methanolic solution (concentration of 10%; *v*/*v* of sample and 90%; *v*/*v* of radicals). Then, the mixtures were left in the darkness at 37 °C. The model that explains the activity of a compound as antiradical is illustrated by Equation (3):

DPPH. + (AH)n →DPPH-H + (A.)n(3)

where AH is an antioxidant that acts as an anti-radical donor of hydrogen atoms, resulting in relatively stable molecular structures and relocation that stops the chain reaction. The newly formed radical (A.) may interact with other molecules to form stable radicals (DPPH-A, A-A). The absorbance was recorded at 517 nm every 15 min during 75 min in total. The results are expressed as mM Trolox/g dry plant.

### 3.4. Evaluation of the Antioxidant Effect of the A. vulneraria Extracts in an Oil-in-Water Emulsion System

#### 3.4.1. Preparation of Emulsion

Alumina was activated in the oven at 200 °C for 24 h, and then cooled in a desiccator until reaching room temperature. Sunflower oil was purchased from a local market and purified two times through alumina in absolute darkness to exclude tocopherols then stored at −80 °C. To prepare the oil-in-water emulsion, 10 % of the purified sunflower oil was added drop by drop to an aqueous mixture containing 1% of Tween-20 and Milli-Q water (Barcelona, Spain) and cooled in an ice bath while sonicating for 10 min. 

#### 3.4.2. Conditioning of the *A. vulneraria* Extracts in Emulsions

The initial emulsion obtained was divided into vials to have at the end 4 samples (each sample was prepared in triplicate) including: a control (emulsion without antioxidant, E-CTR), emulsion containing Gallic acid (E-GA), emulsion containing the *A. vulneraria* leaf extract (E-AVL) and emulsion containing the *A. vulneraria* flower extract (E-AVF). All of the emulsion samples were stored in the oven and allowed to oxidize for 30 days at 35 ± 1 °C in darkness and under constant slow agitation.

#### 3.4.3. Peroxide Value (PV) and pH Measurement

The PV was determined by the ferric thiocyanate method. FeCl_2_ solution (2mM) (made with HCl (1M) and FeCl_2_) and NH_4_SCN solution (2mM) was prepared as the reagent. The assay was performed by diluting a drop of emulsion in the range of 0.007 to 0.0130 g in 1 mL of EtOH 96%. An aliquot from this solution was mixed with 3 mL of EtOH in plastic cuvette and then 75 µL of each reagent was added to the mixture. The absorbance was measured at 500 nm and the results are expressed as meq hydro peroxide/kg emulsion. 

The pH was measured in triplicate each 2 day (pH-meter GLP21, Criston Instruments, Barcelona, Spain) to determine its correlation with the peroxide values. 

#### 3.4.4. Thiobarbituric Acid Reactive Substances (TBARS) Assay

The TBARS assay was determined following the method described in the study of Maqsood and Benjakul [5]. In brief, 0.3 g of each emulsion sample was mixed with 3 mL of TBARS reagent (15% TCA, 0.375% TBA and HCl 2.1%). The samples were mixed in an ultrasonic bath (Prolabo brand equipment, Lutterworth, UK) for 10 min and then placed in a hot water bath at 95 °C for 10 min. After cooling, samples were centrifuged and the absorbance of each supernatant was measured at 531 nm. Results are expressed as mg MDA/kg of emulsion sample.

### 3.5. Evaluation of the Antioxidant Effect of the Powdered A. vulneraria on Raw Ground Meat Quality

#### 3.5.1. Treatment, Preparation and Storage Conditions of the Raw Beef Patties

Three different pieces of ground meat (each piece weighed 300 g), taken from the round part of 3 different cuts, were purchased fresh on 3 different days. Each piece was mixed with salt (1.5% *w*/*w*) and divided into 4 different parts, and then each part was mixed, to homogeneity, with different compounds to finally obtain 4 different treated beef samples: control (without antioxidant), 0.5% T-BHT (treatment with 0.5% synthetic antioxidant (BHT)), 0.5% T-AVL (treatment with 0.5% *A. vulneraria* leaves) and 0.5% T-AVF (treatment with 0.5% *A. vulneraria* flowers). After 3 min of kneading, each blend was flattened and formed into patties of 3–4 g of weight, 4 cm of diameter and 0.5 cm in thickness, using a round cutter. Then, beef patty samples were placed in plastic trays and covered with film and stored in the refrigerator at 4 ± 1 °C for 11 days. The lipid oxidation inhibition and the quality of the beef patties were monitored every 2 days during 11 days. The microbiological analyses were done every 5 days.

#### 3.5.2. Thiobarbituric Acid Reactive Substance Values (TBARS) and pH Measurement

The determination of TBARS in raw beef patties was assessed according to Skowyra et al. [45]. The absorbance of each sample was measured at 531 nm and the results were expressed as mg MDA/kg meat sample. The pH measurement of the beef patties was determined electrometrically every two days using an Orion 3-Star pH Benchtop Meter (Thermo Fisher Scientific, Waltham, MA, USA).

#### 3.5.3. Color Stability Evaluation

The effect of powdered *A. vulneraria* leaves and flowers on the color stability of raw beef patties was evaluated using a reflectance colourimeter Minolta CR-400 (Konica Minolta, Tokyo, Japan) and it was expressed against the scale of L* (lightness), a* (redness) and b* (yellowness) in the CIELab colour space system. Before each measuring session (light source of D65 and 10° standard observer), the instrument was calibrated (white reference: *Y* = 93.8; *x* = 0.315; *y* = 0.332). Three measurements from three different locations on the raw beef patties surface were taken on each day of analysis. Fat zones were avoided in order to obtain correct measurements.

#### 3.5.4. Antioxidant Capacity Measurement (AOC)

AOC of meat samples was determined by the hydrophilic and lipophilic FRAP assays. The preparation of the samples was conducted as described by Gallego et al. [49] using hydrophilic (distilled water) and lipophilic (acetone, ethanol and distilled water (5:4:1; *v*/*v*/*v*)) solvents, used for the extraction of hydrophilic and lipophilic antioxidants, respectively. The different extracts were used to perform the FRAP assay as described previously. The results were expressed as µmol eq Trolox/mL.

#### 3.5.5. Sensory Analysis

The sensory characteristics of beef pattie samples were evaluated by the discriminative triangle test. A non-trained panel composed of 31 students between 18 and 22 years old were selected from the School of Industrial Engineering of Barcelona (ETSEIB). The different beef pattie samples (Control, T-AVL and T-AVF) were cooked in a Hamburger Grill (Tristar, GR-2843, Barcelona, Spain ) at full power for 3 min and presented directly to the panelists. In the triangle test, each panelist chose the odd one of the three samples (two similar samples and a different one). Water, apples and biscuits were provided for cleaning the palate after tasting each sample.4.7. Data analysis

Analysis was carried out in triplicate (*n* = 3) and standard deviations (SD) were calculated. All the data were analyzed by the MINITAB software program (Version 18, München, Germany) using Tukey′s test. The significance of differences (*p* < 0.05) between mean values was determined by the one-way analysis of variance (ANOVA).

## 4. Conclusions

The results obtained in this study showed that the leaves and flowers of *A. vulneraria* are rich in phenolic compounds and have significant antioxidant activity with significant differences between the two parts, and the flowers presented the best results. The antioxidant effect of the *A. vulneraria* leaves and flowers in a model food emulsion was investigated for the first time in this study. The flowers had a more notable effect on oxidation in the oil-in-water emulsion system than the leaves, as they had the most effective antioxidant effect against lipid oxidation in raw beef patties during refrigerated storage. In conclusion, the use of the *A. vulneraria* as a natural additive in food, cosmetic and pharmaceutical products is an effective strategy to improve their nutritional and medical value while ensuring consumer safety.

## Figures and Tables

**Table 1:** TPC (Total Polyphenol Content) and TFC (Total Flavonoid Content) results of the leaf and flower extracts of *A. vulneraria* in 50% aqueous ethanol.

Samples	TPC (mg GAE/g Dry Plant)	TFC (mg QE/g Dry Plant)
Leaf	82.86 ± 1.22 ^b^	20.14 ± 0.18 ^b^
Flower	134.31 ± 1.64 ^a^	33.58 ± 4.07 ^a^

Results represent the mean of three replicates (*n* = 3) and are expressed as mean value ± SD; different letters indicate significant differences in each column at *p* < 0.05. GAE (Gallic Acid Equivalent), QE (Quercetin Equivalent).

**Table 2:** Antioxidant activity of *A. vulneraria* leaf and flower extracts extracted with 50% aqueous ethanol.

Samples	FRAP (mM Trolox/g Dry Plant)	ORAC (mM Trolox/g Dry Plant)	TEAC (mM Trolox/g Dry Plant)	DPPH (mM Trolox/g Dry Plant)
Leaves	1.90 ± 0.01 ^b^	0.98 ± 0.003 ^b^	0.82 ± 0.002 ^b^	0.33 ± 1.08 ^b^
Flowers	3.30 ± 0.01 ^a^	1.64 ± 0.07 ^a^	1.42 ± 0.01 ^a^	0.64 ± 0.96 ^a^

Results represent the mean of three replicates (*n* = 3) and are expressed as mean value ± SD; different letters indicate significant differences in each column at *p* < 0.05.

**Table 3:** The pH values of raw beef patties formulated with powdered leaves and flowers of *A. vulneraria* during storage at 4 ± 1 °C CTR (Control sample without antioxidant), BHT (treatment with synthetic antioxidant (Butylated hydroxytoluene)), T-AVL (treatment with *A. vulneraria* leaf), T-AVF (treatment with *A. vulneraria* flower).

Days of Storage	1	2	4	6	8	11
CTR	5.86 ± 0.03 ^a^	5.89 ± 0.02 ^a^	5.92 ± 0.01 ^a^	5.97 ± 0.02 ^a^	6.01 ± 0.02 ^a^	6.19 ± 0.03 ^a^
BHT	5.69 ± 0.01 ^c^	5.70 ± 0.11 ^b^	5.72 ± 0.02 ^c^	5.74 ± 0.01 ^b^	5.77 ± 0.01 ^c^	5.78 ± 0.02 ^c^
T-AVL	5.76 ± 0.01 ^b^	5.78 ± 0.03 ^ab^	5.82 ± 0.03 ^b^	5.87 ± 0.1 ^ab^	5.93 ± 0.1 ^ab^	6.01 ± 0.05 ^b^
T-AVF	5.71 ± 0.01 ^c^	5.74 ± 0.02 ^b^	5.79 ± 0.05 ^bc^	5.81 ± 0.03 ^ab^	5.84 ± 0.03 ^bc^	5.86 ± 0.02 ^c^

Results represent the mean of three replicates (*n* = 3) and are expressed as mean value ± SD, different letters in the same day indicate significant difference between samples at *p* < 0.05.

**Table 4:** Color characteristics of raw beef patties formulated with powdered leaves and flowers of *A. vulneraria* during storage at 4 ± 1 °C CTR (Control sample without antioxidant), BHT (treatment with synthetic antioxidant (Butylated hydroxytoluene)), T-AVL (treatment with *A. vulneraria* leaf), T-AVF (treatment with 0.5% *A. vulneraria* flower).

Trait	Days	CTR	BHT	T-AVL	T-AVF
Redness (a*)	1	36.67 ± 2.37 ^a^	52.32 ± 0.98 ^b^	43.75 ± 4.28 ^ab^	49.46 ± 0.37 ^ab^
	2	33.80 ± 0.37 ^a^	49.26 ± 2.56 ^b^	39.96 ± 1.23 ^ab^	48.01 ± 0.05 ^ab^
	4	27.95 ± 0.71 ^a^	48.91 ± 1.63 ^b^	38.21 ± 0.95 ^ab^	43.07 ± 0.03 ^ab^
	6	27.76 ± 0.84 ^a^	48.77 ± 0.69 ^b^	38.02 ± 0.47 ^ab^	41.80 ± 1.85 ^ab^
	8	25.14 ± 0.12 ^a^	43.68 ± 0.81 ^b^	34.09 ± 0.83 ^ab^	31.22 ± 0.18 ^ab^
	11	24.89 ± 1.23 ^a^	37.31 ± 1.09 ^b^	26.84 ± 1.46 ^ab^	25.74 ± 1.03 ^ab^
Yellowness (b*)	1	10.30 ± 0.17 ^a^	15.35 ± 0.02 ^b^	10.45 ± 1.24 ^a^	14.34 ± 0.57 ^b^
	2	10.28 ± 1.92 ^a^	14.63 ± 1.19 ^b^	9.93 ± 2.51 ^a^	13.32 ± 0.11 ^b^
	4	9.58 ± 1.93 ^a^	13.43 ± 0.41 ^b^	9.88 ± 0.15 ^a^	13.29 ± 0.04 ^b^
	6	9.46 ± 0.55 ^a^	13.35 ± 0.10 ^b^	8.78 ± 0.04 ^a^	12.11 ± 0.24 ^b^
	8	5.63 ± 0.07 ^a^	11.79 ± 1.24 ^b^	8.10 ± 1.00 ^a^	10.79 ± 0.53 ^b^
	11	5.17 ± 1.03 ^a^	11.13 ± 0.46 ^b^	5.52 ± 0.95 ^a^	9.92 ± 0.14 ^b^
Lightness (L*)	1	56.18 ± 0.14 ^a^	70.80 ± 2.54 ^ab^	68.36 ± 2.09 ^ab^	70.99 ± 2.19 ^b^
	2	55.42 ± 3.07 ^a^	61.86 ± 1.62 ^ab^	65.07 ± 2.12 ^ab^	65.09 ± 4.12 ^b^
	4	55.30 ± 0.25 ^a^	60.61 ± 0.33 ^ab^	61.52 ± 1.80 ^ab^	63.70 ± 3.63 ^b^
	6	55.04 ± 1.60 ^a^	56.78 ± 2.78 ^ab^	58.64 ± 1.97 ^ab^	62.18 ± 0.40 ^b^
	8	52.59 ± 0.50 ^a^	56.65 ± 1.80 ^ab^	55.54 ± 1.72 ^ab^	59.53 ± 0.23 ^b^
	11	43.63 ± 2.98 ^a^	49.39 ± 0.04 ^ab^	49.74 ± 0.21 ^ab^	55.61 ± 4.65 ^b^

Results represent the mean of three replicates (*n* = 3) and are expressed as mean value ± SD; different letters in the same day indicate significant difference between samples at *p* < 0.05.

**Table 5:** Sensorial analysis of beef patties formulated with powdered leaves and flowers of *A. vulneraria* T-AVL (treatment with *A. vulneraria* leaf), T-AVF (treatment with *A. vulneraria* flower).

Meat Samples	Number of Assessors			Number of Smokers/Non-Smokers		Odd Samples Identified		Level of Significance
	Male	Female	Total	Smoker	Non-Smoker	Correct (+)	Incorrect (-)	
T-AVL	18	7	25	1	24	23	2	0.1%
T-AVF	18	12	30	3	27	23	7	0.1%

## References

[1] Ouerghemmi I., Bettaieb Rebey I., Rahali F.Z., Bourgou S., Pistelli L., Ksouri R., Marzouk B., Saidani Tounsi M. (2017). Antioxidant and antimicrobial phenolic compounds from extracts of cultivated and wild-grown Tunisian *Ruta chalepensis*. J. Food Drug Anal..

[2] Gallego M.G., Rodriguez T., Rodriguez I., Almajano M.P. (2016). Analytical Characterization of Polyphenols from Tara and *Caesalpinia decapetala* as Stabilizers of O/W Emulsions. J. Food Sci..

[3] Kchaou W., Abbès F., Mansour R.B., Blecker C., Attia H., Besbes S. (2016). Phenolic profile, antibacterial and cytotoxic properties of second grade date extract from Tunisian cultivars (*Phoenix dactylifera* L.). Food Chem..

[4] Zilani M.N.H., Sultana T., Asabur Rahman S.M., Anisuzzman M., Islam M.A., Shilpi J.A., Hossain M.G. (2017). Chemical composition and pharmacological activities of *Pisum sativum*. BMC Complement. Altern. Med..

[5] Maqsood S., Benjakul S. (2010). Comparative studies of four different phenolic compounds on in vitro antioxidative activity and the preventive effect on lipid oxidation of fish oil emulsion and fish mince. Food Chem..

[6] Pisoschi A.M., Pop A. (2015). The role of antioxidants in the chemistry of oxidative stress: A review. Eur. J. Med. Chem..

[7] Gallego G., Hakkarainen M., Almajano M.P. (2016). Stability of O/W emulsions packed with PLA film with incorporated rosemary and thyme. Eur. Food Res. Technol..

[8] Lin M.-J., Chang S.-C., Jea Y.-S., Liao J.-W., Fan Y.-K., Lee T.-T. (2016). In vitro antioxidant capability and performance assessment of White *Roman goose* supplemented with dried *Toona sinensis*. J. Appl. Anim. Res..

[9] Wardhani D.H., Fuciños P., Vázquez J.A., Pandiella S.S. (2013). Inhibition kinetics of lipid oxidation of model foods by using antioxidant extract of fermented soybeans. Food Chem..

[10] Balasundram N., Sundram K., Samman S. (2006). Phenolic compounds in plants and agri-industrial by-products: Antioxidant activity, occurrence, and potential uses. Food Chem..

[11] Wang Y.C., Chuang Y.C., Ku Y.H. (2007). Quantitation of bioactive compounds in citrus fruits cultivated in Taiwan. Food Chem..

[12] Palici I.F., Liktor-Busa E., Zupkó I., Touzard B., Chaieb M., Urbán E., Hohmann J. (2015). Study of in vitro antimicrobial and antiproliferative activities of selected Saharan plants. Acta Biol. Hung..

[13] Mhalla D., Bouaziz A., Ennouri K., Chawech R., Smaoui S., Jarraya R., Tounsi S., Trigui M. (2017). Antimicrobial activity and bioguided fractionation of Rumex tingitanus extracts for meat preservation. Meat Sci..

[14] Gepts P., Beavis W.D., Brummer E.C., Shoemaker R.C., Stalker H.T., Weeden N.F., Young N.D. (2005). Legumes as a model plant family. Genomics for food and feed report of the Cross-Legume Advances Through Genomics Conference. Plant Physiol..

[15] Graham P.H., Vance C.P. (2014). Update on Legume Utilization Legumes: Importance and Constraints to Greater Use. Plant Physiol..

[16] Nartowska J., Wawer I., Strzelecka H. (2001). Triterpenoid sapogenin from *Anthyllis vulneraria L.*. Acta Pol. Pharm. Drug Res..

[17] Halabalaki M., Urbain A., Paschali A., Mitakou S., Tillequin F., Skaltsounis A.L. (2011). Quercetin and kaempferol 3-*O*-[α-l-rhamnopyranosyl-(→2)-α-l-arabinopyranoside]-7-*O*-α-l-rhamnopyranosides from *Anthyllis hermanniae*: Structure determination and conformational studies. J. Nat. Prod..

[18] Suganda A.G., Amoros M., Girre L., Fauconnier B. (1983). Purifies De Plantes Indigenes Francaises Sur La Multiplication De L ’ Herpesvirus Humain 1 Et Du. J. Nat. Prod..

[19] Godevac D., Zdunić G., Šavikin K., Vajs V., Menković N. (2008). Antioxidant activity of nine Fabaceae species growing in Serbia and Montenegro. Fitoterapia.

[20] Csepregi R., Bencsik T., Papp N. (2016). Examination of secondary metabolites and antioxidant capacity of *Anthyllis vulneraria, Fuchsia* sp., *Galium mollugo* and *Veronica beccabunga*. Acta Biol. Hung..

[21] Tusevski O., Kostovska A., Iloska A., Trajkovska L., Simic S.G. (2014). Phenolic production and antioxidant properties of some Macedonian medicinal plants. Cent. Eur. J. Biol..

[22] Son S.Y., Kim N.K., Lee S., Singh D., Kim G.R., Lee J.S., Yang H.S., Yeo J., Lee S., Lee C.H. (2016). Metabolite fingerprinting, pathway analyses, and bioactivity correlations for plant species belonging to the Cornaceae, Fabaceae, and Rosaceae families. Plant Cell Rep..

[23] Skowyra M., Gallego G. (2013). Antioxidant properties of aqueous and ethanolic extracts of tara (*Caesalpinia spinosa*) pods in vitro and in model food emulsions. J. Sci Food Agric..

[24] Ksouri R., Megdiche W., Falleh H., Trabelsi N., Boulaaba M., Smaoui A., Abdelly C. (2008). Influence of biological, environmental and technical factors on phenolic content and antioxidant activities of Tunisian halophytes. Comptes Rendus Biologies.

[25] Santas J., Carbó R., Gordon M.H., Almajano M.P. (2008). Comparison of the antioxidant activity of two Spanish onion varieties. Food Chem..

[26] Gallego M.G., Gordon M.H., Segovia F.J., Skowyra M., Almajano M.P. (2013). Antioxidant properties of three aromatic herbs (rosemary, thyme and lavender) in oil-in-water emulsions. J. Am. Oil Chem. Soc..

[27] Pahua-Ramos M.E., Ortiz-Moreno A., Chamorro-Cevallos G., Hernández-Navarro M.D., Garduño-Siciliano L., Necoechea-Mondragón H., Hernández-Ortega M. (2012). Hypolipidemic Effect of Avocado (*Persea americana Mill*) Seed in a Hypercholesterolemic Mouse Model. Plant Foods Hum. Nutr..

[28] Cheung L.M., Cheung P.C.K., Ooi V.E.C. (2003). Antioxidant activity and total phenolics of edible mushroom extracts. Food Chem..

[29] Ye F., Liang Q., Li H., Zhao G. (2015). Solvent effects on phenolic content, composition, and antioxidant activity of extracts from florets of sunflower (*Helianthus annuus L*.). Ind. Crops Prod..

[30] Saha J., Debnath M., Saha A., Ghosh T., Sarkar P.K. (2011). Response surface optimisation of extraction of antioxidants from strawberry fruit, and lipid peroxidation inhibitory potential of the fruit extract in cooked chicken patties. J. Sci. Food Agric..

[31] Fernández-Agulló A., Pereira E., Freire M.S., Valentão P., Andrade P.B., González-álvarez J., Pereira J.A. (2013). Influence of solvent on the antioxidant and antimicrobial properties of walnut (*Juglans regia* L.) green husk extracts. Ind. Crops Prod..

[32] Veloz-García R.A., Marín-Martínez R., Veloz-Rodríguez R., Muñoz-Sánchez C.I., Guevara-Olvera L., Miranda-López R., González-Chavira M.M., Torres-Pacheco I., Guzmán-Maldonado S.H., Cardador-Martínez A. (2004). Antimutagenic and antioxidant activities of cascalote (*Caesalpinia cacalaco*) phenolics. J. Sci. Food Agric..

[33] Feregrino-Pérez A.A., Torres-Pacheco I., Vargas-Hernández M., Munguía-Fragozo P.V., Loarca-Piña G.F., Mendoza-Díaz S.O., Ocampo-Velázquez R.V., Rico-García E., Guevara-Gónzalez R.G. (2011). Antioxidant and antimutagenic activities of *Acacia pennatula* pods. J. Sci. Ind. Res..

[34] Karakoca K., Asan-Ozusaglam M., Cakmak Y.S., Teksen M. (2015). Phenolic Compounds, biological and antioxidant activities of *onobrychis armena Boiss. & Huet* flower and root extracts. Chiang Mai J. Sci..

[35] Kenny O., Smyth T.J., Hewage C.M., Brunton N.P. (2013). Antioxidant properties and quantitative UPLC-MS analysis of phenolic compounds from extracts of fenugreek (*Trigonella foenum-graecum*) seeds and bitter melon (*Momordica charantia*) fruit. Food Chem..

[36] Skowyra M., Gallego M., Segovia F., Almajano M. (2014). Antioxidant Properties of *Artemisia annua* Extracts in Model Food Emulsions. Antioxidants.

[37] Azman N., Segovia F., Martínez-Farré X., Gil E., Almajano M. (2014). Screening of Antioxidant Activity of *Gentian Lutea* Root and Its Application in Oil-in-Water Emulsions. Antioxidants.

[38] Segovia Gómez F., Almajano Pablos M.P. (2016). Pineapple Waste Extract for Preventing Oxidation in Model Food Systems. J. Food Sci..

[39] Davey M.W., Stals E., Panis B., Keulemans J., Swennen R.L. (2005). High-throughput determination of malondialdehyde in plant tissues. Anal. Biochem..

[40] Frankel E.N., Huang S.W., Aeschbach R., Prior E. (1996). Antioxidant Activity of a Rosemary Extract and Its Constituents, Carnosic Acid, Carnosol, and Rosmarinic Acid, in Bulk Oil and Oil-in-Water Emulsion. J. Agric. Food Chem..

[41] Decker E.A., Warner K., Richards M.P., Shahidi F. (2005). Measuring antioxidant effectiveness in food. J. Agric. Food Chem..

[42] Sun Y.E., Wang W.D., Chen H.W., Li C. (2011). Autoxidation of unsaturated lipids in food emulsion. Crit. Rev. Food Sci. Nutr..

[43] Leygonie C., Britz T.J., Hoffman L.C. (2012). Impact of freezing and thawing on the quality of meat: Review. Meat Sci..

[44] Riazi F., Zeynali F., Hoseini E., Behmadi H., Savadkoohi S. (2016). Oxidation phenomena and color properties of grape pomace on nitrite-reduced meat emulsion systems. Meat Sci..

[45] .Skowyra M., Janiewicz U., Salejda A.M., Krasnowska G., Almajano M.P. (2015). Effect of tara (*Caesalpinia spinosa*) pod powder on the oxidation and colour stability of pork meat batter during chilled storage. Food Technol. Biotechnol..

[46] Azman A., Gallego M., Julià L., Fajari L., Almajano M.P. (2015). The effect of c*onvolvulus arvensis* dried extract as a potential antioxidant in food models. Antioxidants.

[47] Muela E., Sañudo C., Campo M.M., Medel I., Beltrán J.A. (2010). Effect of freezing method and frozen storage duration on instrumental quality of lamb throughout display. Meat Sci..

[48] Li M., Li X., Xin J., Li Z., Li G., Zhang Y., Du M., Shen Q.W., Zhang D. (2017). Effects of protein phosphorylation on color stability of ground meat. Food Chem..

[49] Gallego M.G., Gordon M.H., Segovia F.J., Almajano M.P. (2015). Caesalpinia decapetala Extracts as Inhibitors of Lipid Oxidation in Beef Patties. Molecules.

[50] Hawashin M.D., Al-Juhaimi F., Ahmed I.A.M., Ghafoor K., Babiker E.E. (2016). Physicochemical, microbiological and sensory evaluation of beef patties incorporated with destoned olive cake powder. Meat Sci..

[51] Mancini R.A., Hunt M.C. (2005). Current research in meat color. Meat Sci..

[52] Muthukumar M., Naveena B.M., Vaithiyanathan S., Sen A.R., Sureshkumar K. (2012). Effect of incorporation of Moringa oleifera leaves extract on quality of ground pork patties. J. Food Sci. Technol..

[53] Esmer O.K., Irkin R., Degirmencioglu N., Degirmencioglu A. (2011). The effects of modified atmosphere gas composition on microbiological criteria, color and oxidation values of minced beef meat. Meat Sci..

[54] Pękal A., Pyrzynska K. (2014). Evaluation of Aluminium Complexation Reaction for Flavonoid Content Assay. Food Anal. Methods..

[55] Shalaby E.A., Shanab S.M.M. (2013). Comparison of DPPH and ABTS assays for determining antioxidant potential of water and methanol extracts of *Spirulina platensis*. Indian J. Geomarine Sci..

